# Supplementary material for: A nucleotide resolution map of Top2-linked DNA breaks in the yeast and human genome
Source: Nat Commun. 2019 Oct 24;10:4846. doi: 10.1038/s41467-019-12802-5 (PMC6813358; doi:10.1038/s41467-019-12802-5)
Supplement: Supplementary file 1 — Supplementary Information [file 41467_2019_12802_MOESM1_ESM.pdf]

Supplementary information for:

A nucleotide resolution map of Top2-linked DNA breaks in the yeast and human genome

Gittens et al. 2019

Supplementary Figures 1-13
Supplementary Tables 1-6

Supplementary Figure 1

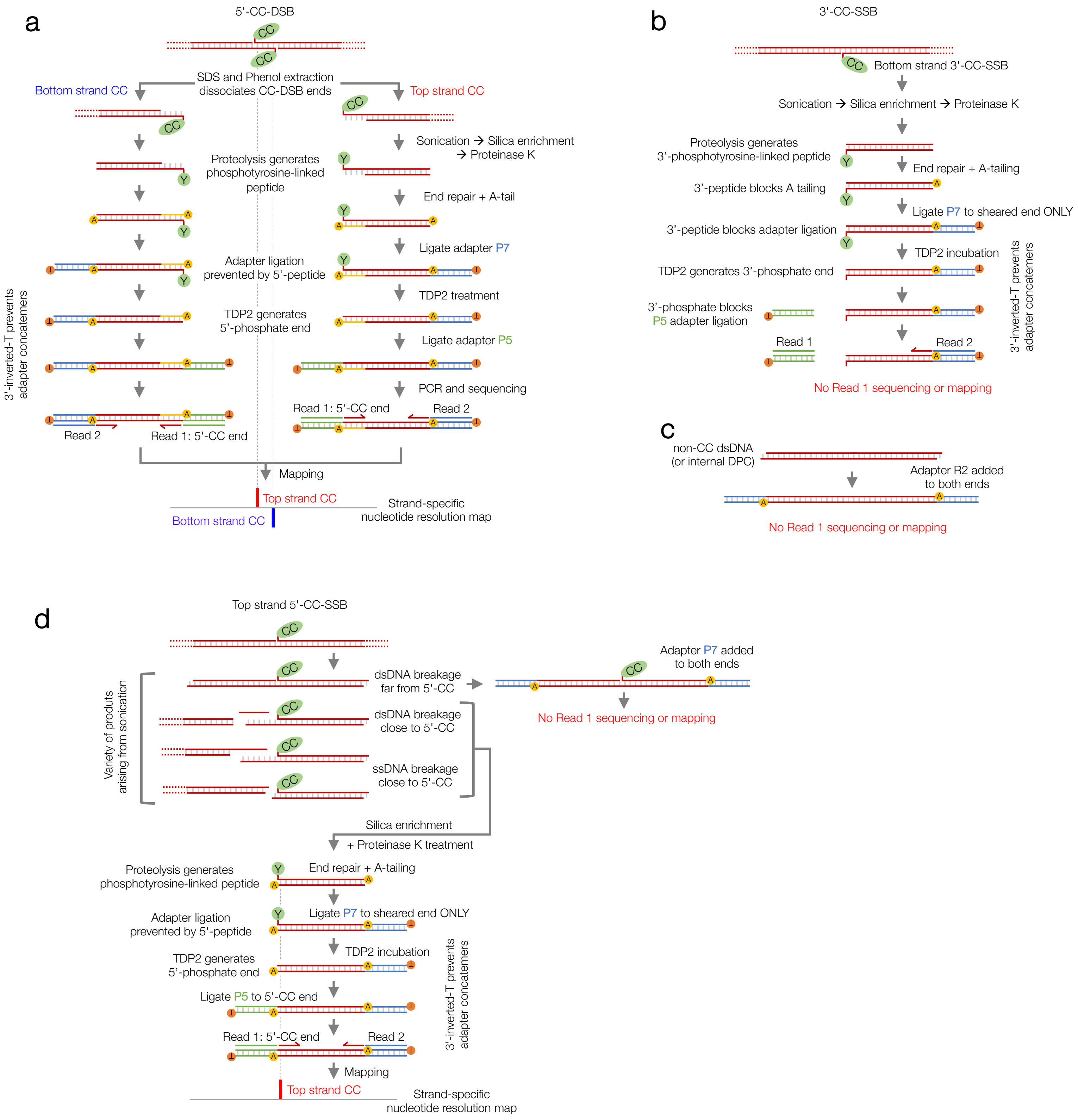

Supplementary Figure 1. CC-seq library preparation steps selectively capture 5'-CC-DSBs and 5'-CC-SSBs, whilst excluding contaminant non-CC dsDNA, internal DNA-protein crosslinks (DPCs), and 3'-CC-SSBs.

a, Diagram showing how a 5'-CC-DSB receives adapter P7 on the sonicated termini, and P5 on the CC termini, resulting in sequenceable molecules.

b, Diagram showing how a 3'-CC-SSB receives adapter P7 on the sonicated terminus, and no adapter on the CC terminus, resulting in a non-sequenceable molecule.

c, Diagram showing how non-CC dsDNA and/or molecules with an internal DNA-protein crosslink (DPC) each receive adapter P7 on both termini, resulting in non-sequenceable molecules.

d, Diagram showing how a 5'-CC-SSB receives adapter P7 on the sonicated terminus, and P5 on the CC terminus, which is converted to a DSB end via sonication, resulting in a sequenceable molecule.

Supplementary Figure 2

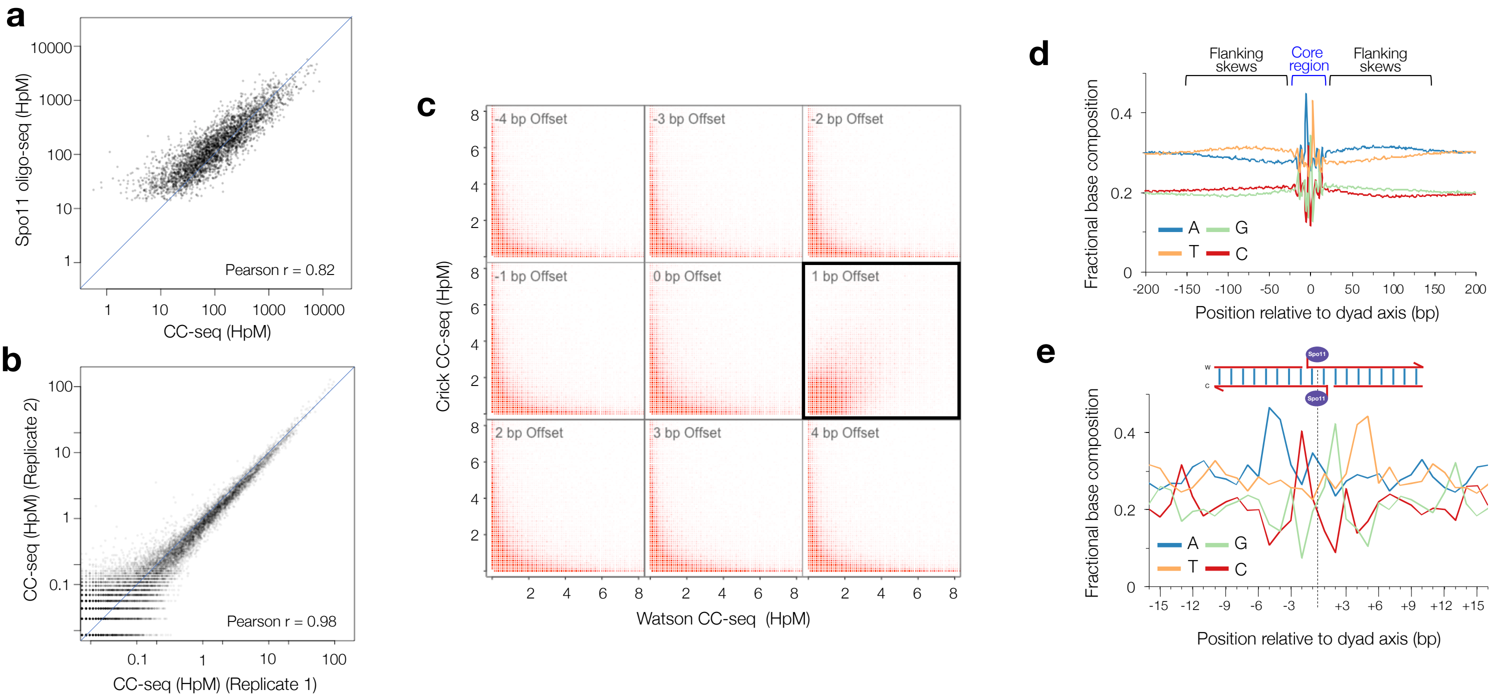

Supplementary Figure 2. CC-seq maps covalent Spo11-linked DNA breaks in *S. cerevisiae* meiosis with nucleotide precision

a, Correlation of CC-seq and oligo-seq within annotated Spo11 hotspots[28].

b, Correlation of 500 bp binned Spo11 maps from two representative replicates.

c, Correlation of Spo11 cleavages on Watson and Crick strand when offset by 1 bp (boxed), relative to other offsets from -4 to +4 bp.

d-e, Average nucleotide composition over a ±200 bp (**d**) or 'core' ±15 bp (**e**) window centred on the inferred dyad axis of Spo11 DSBs identified by CC-seq. Bases reported are for the top strand only. The core region is likely to be the bases directly influencing Spo11 binding and/or catalysis[28]. Weak, rotationally symmetrical ~150 bp wide flanking skews are likely to be caused by chromatin structure adjacent to preferred Spo11-DSB sites.

Supplementary Figure 3

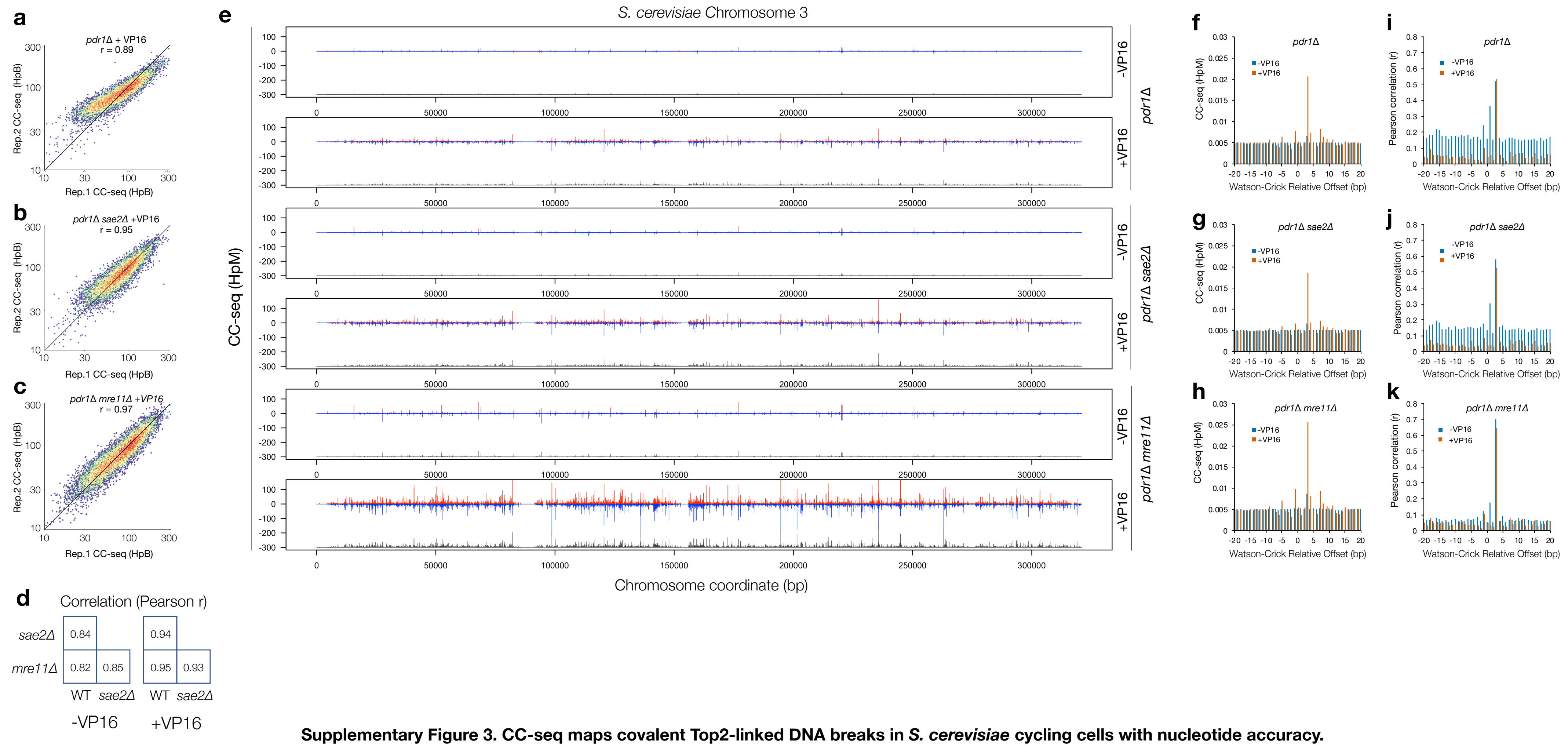

Supplementary Figure 3. CC-seq maps covalent Top2-linked DNA breaks in *S. cerevisiae* cycling cells with nucleotide accuracy.

a-c, Correlation of 1 Kbp binned Top2 CC maps from *pdr1Δ* (A), *pdr1Δsae2Δ* (B) and *pdr1Δmre11Δ* (C) cells treated with VP16.

d, Pairwise Pearson correlation values for 100 bp binned Top2 CC maps from all assayed conditions. Each condition is a pool of two biological replicates.

e, Nucleotide-resolution vegetative *S. cerevisiae* Top2 CC map of chromosome 3 for all assayed conditions. Each condition is a pool of two biological replicates.

f-h, The normalised number of Top2 CCs retained in the *pdr1Δ* (**f**), *pdr1Δsae2Δ* (**g**) and *pdr1Δmre11Δ* (**h**) cells treated ±VP16, after filtering to include only sites offset by the given number of base pairs. All data were normalised over a -100 to +100 bp window.

i-k, Pearson correlation (r) of Top2 CC-seq signal on Watson and Crick strands, offset by the indicated distance in *pdr1Δ* (**i**), *pdr1Δsae2Δ* (**j**) and *pdr1Δmre11Δ* (**k**) cells treated ±VP16.

Supplementary Figure 4

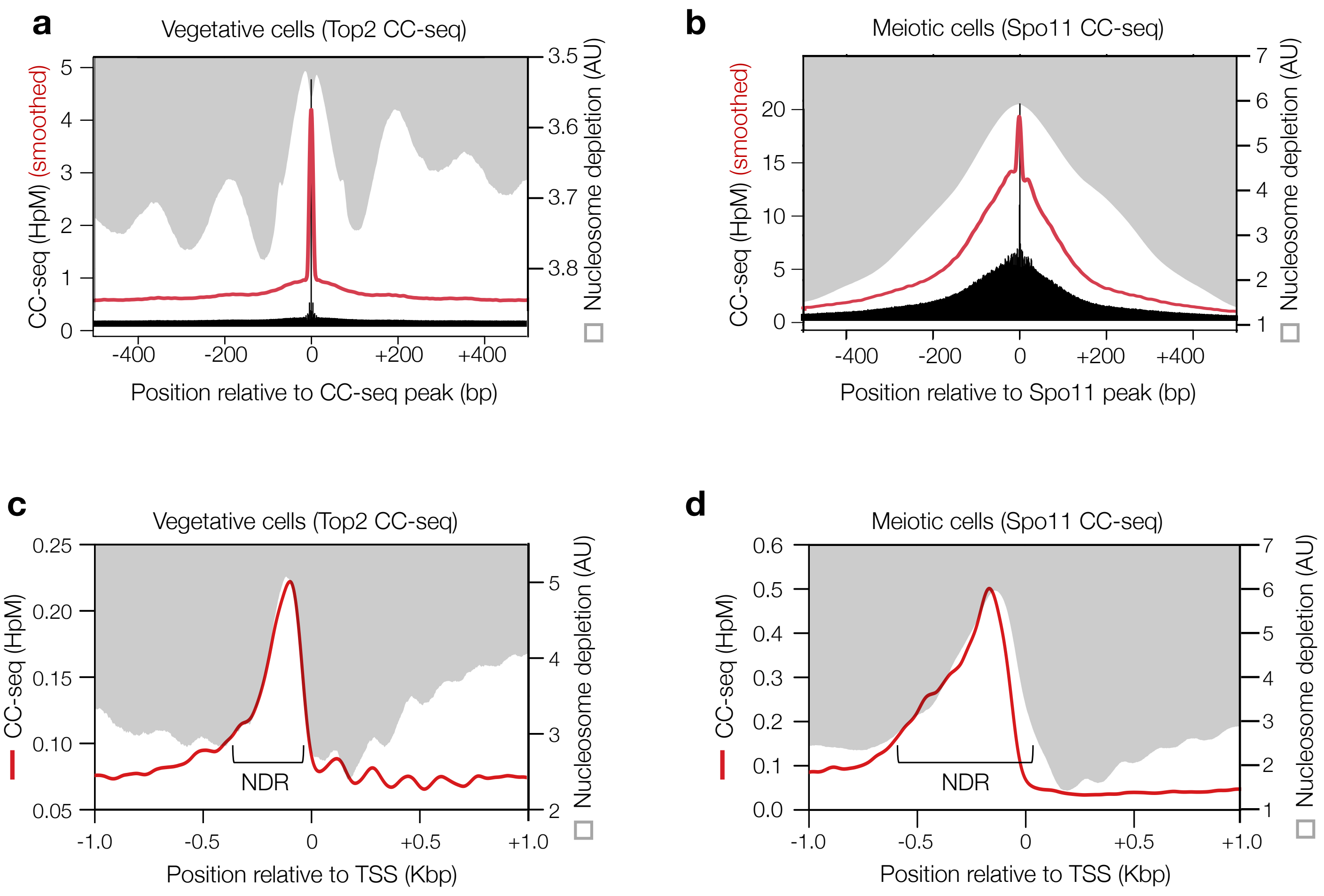

Supplementary Figure 4. Top2 CC and Spo11 CC anticorrelate with nucleosome occupancy both globally and at TSSs.

a-b, Top2 CCs (**a**), and Spo11 CCs (**b**), mapped by CC-seq (raw=black, or smoothed=red) both anticorrelate with nucleosome occupancy measured by MNase-seq[37] (grey).

c-d, Top2 CC (**c**), and Spo11 CC (**d**) signals mapped by CC-seq (red) and nucleosome occupancy (grey) were aggregated centred on transcription start sites (TSSs) and smoothed with a 50 bp hann window. MNase-seq data[37] in (**a-d**) is inverted to emphasise spatial relationship between CC-seq and nucleosome depletion (white). Averaged meiotic (**d**) nucleosome depleted regions (NDRs) are wider than in vegetative cells (**c**), a relationship that correlates with averaged CC-seq profiles.
HpM = Hits per million mapped reads per base pair.

Supplementary Figure 5

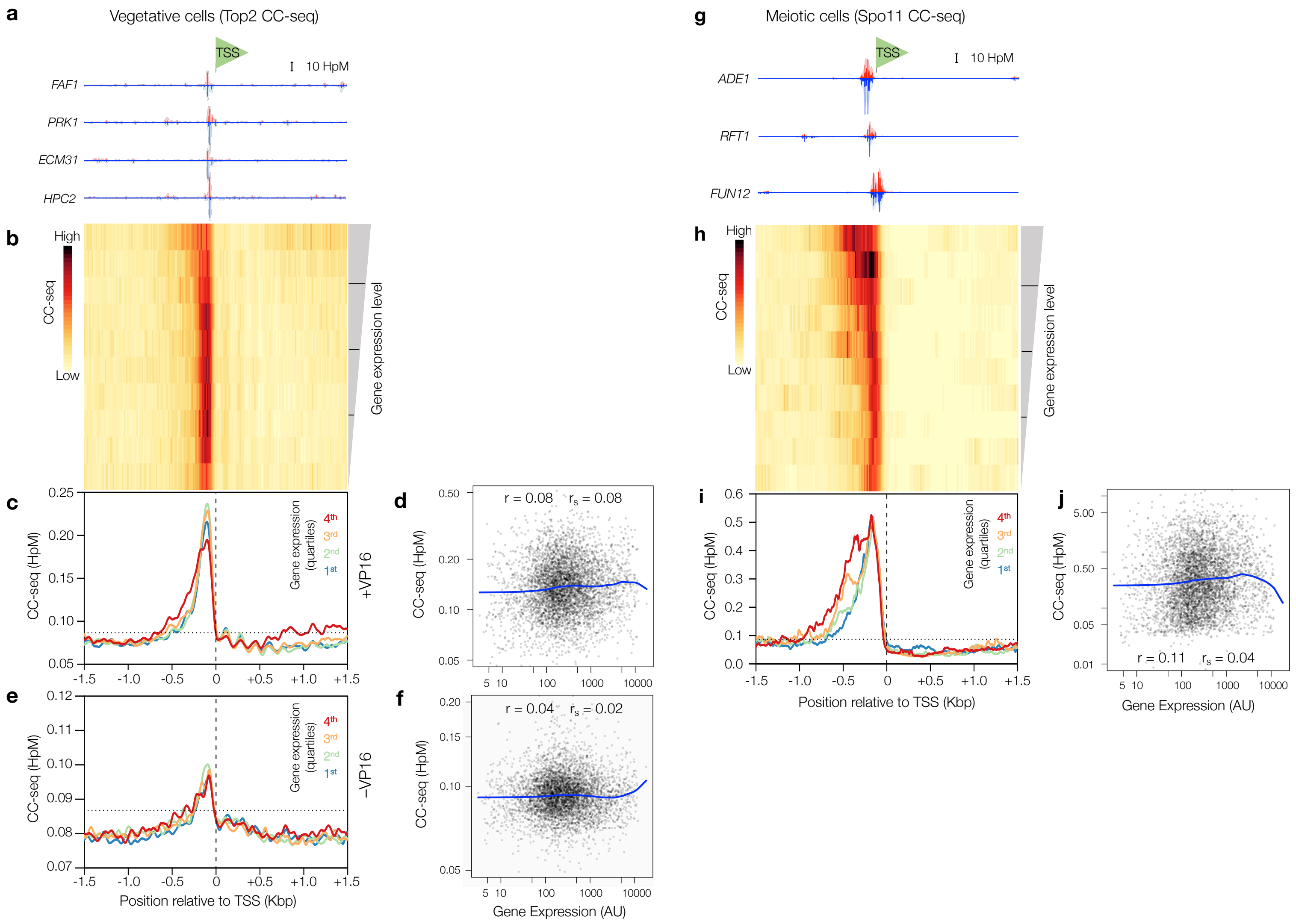

Supplementary Figure 5. Top2 CC and Spo11 CC in *S. cerevisiae* are not quantitatively correlated with gene expression

a-c, Aggregation of Top2 CCs in a 3 Kbp window centred on orientated TSSs in VP16-treated vegitive *pdr1Δ S. cerevisiae* cells. Four example TSS loci are shown orientated in the 5'-3' direction (**a**). A heatmap of all TSS loci in the *S. cerevisiae* genome, with 10 aggregated rows stratified by the strength of downstream gene expression[40]. The colour scale indicates average Top2 CC density in each 15 bp bin (**b**). Loci were also stratified into quartiles of gene expression, and the average Top2 CC distribution in each quartile plotted. Horizontal line is the genome mean. Vertical line is the TSS (**c**).

d, Quantitative correlation between vegetative Top2 CC-seq (500 bp window directly upstream of TSS) and expression of downstream gene. Blue line = lowess curve. Pearson r and Spearman r_s values were calculated between CC-seq and log-transformed Expression.

e-f, As for (**c-d**), but vegetative Top2 CC-seq samples not treated with VP16.

g-j, As for (**a-d**), but meiotic Spo11 CC-seq analysis correlated against meiotic gene expression[40].

Supplementary Figure 6

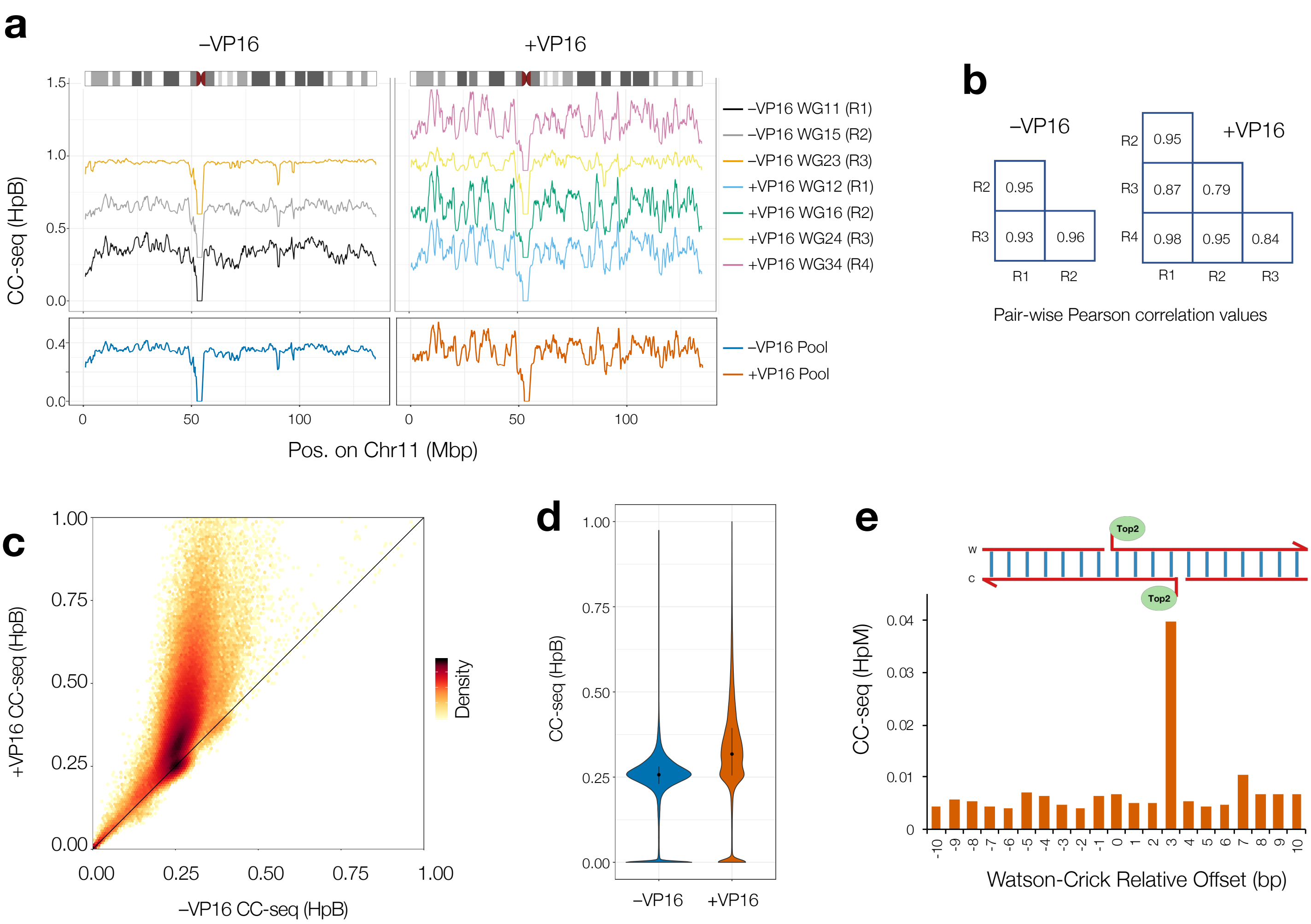

Supplementary Figure 6. CC-seq maps of TOP2-linked DNA breaks in Human cells are enriched by VP16, and show high reproducibility and nucleotide accuracy

a, Broad-scale TOP2 CC-seq maps in individual biological replicates of human RPE-1 cells ±VP16. Raw data were binned at 100 Kbp prior to plotting. Each plot is offset on the y-axis by +0.3 HpB.

b, Replicate-to-replicate Pearson correlation values (r) for 10 Kbp binned TOP2 CC-seq maps of RPE-1 cells ±VP16.

c, Scatter plot of -VP16 and +VP16 TOP2 CC-seq maps binned at 10 Kbp resolution. Data were first scaled according to the estimated noise fraction, and are presented in a hexagonal-binned format, where the density of overplotting is indicated by the colour scale.

d, Violin plots of TOP2 CC-seq maps ±VP16 binned at 10 Kbp resolution. The inner black bar and black dot indicate the interquartile range and median.

e, The normalised number of TOP2 CCs retained in the CC-seq maps in RPE-1 cells +VP16 after filtering to include only sites offset by the given number of base pairs. Data were normalised over a ±100 bp window.

HpM, HpB = Hits per million (or billion) mapped reads per base pair.

Supplementary Figure 7

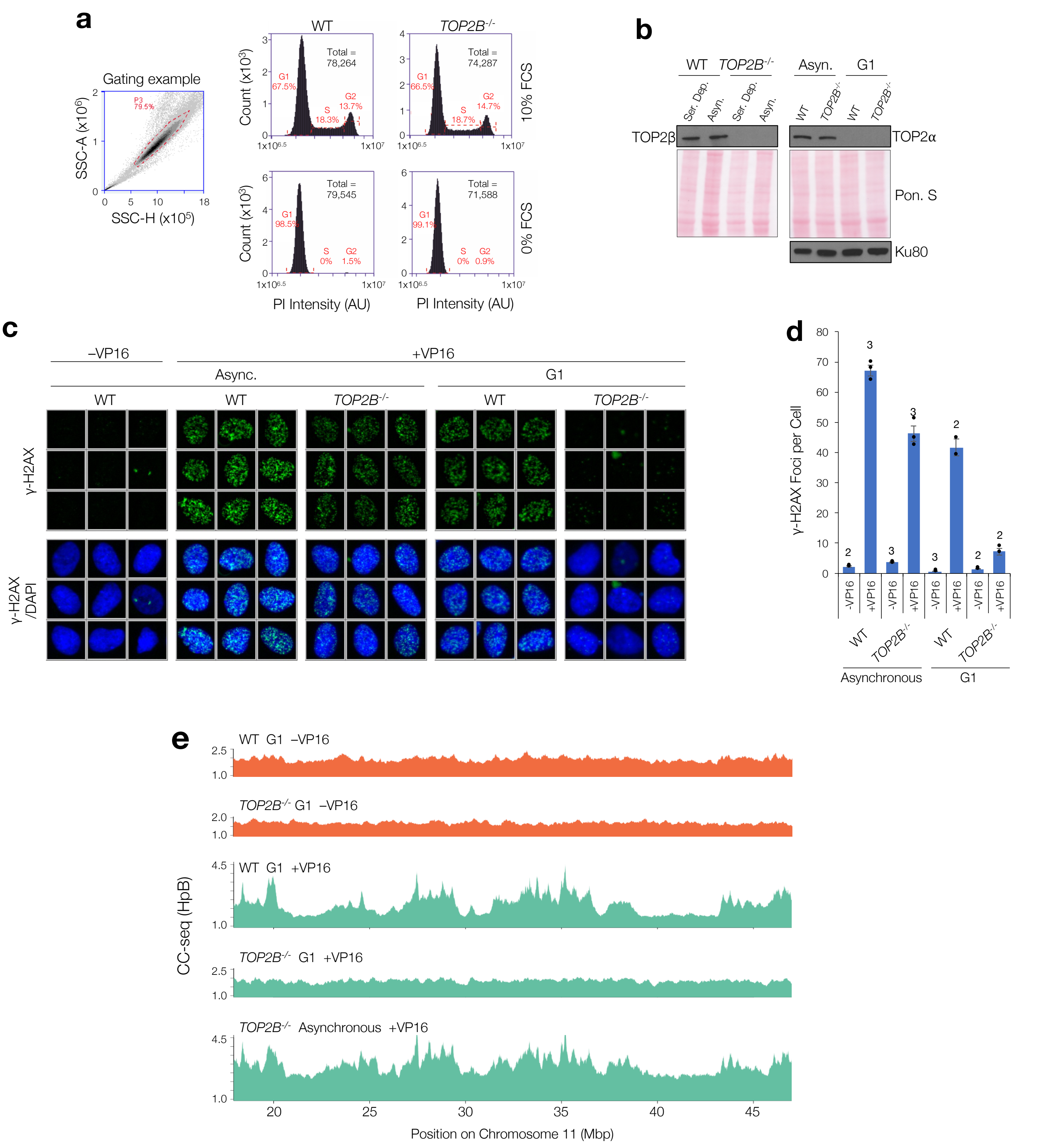

Supplementary Figure 7. CC-seq signal is TOP2-dependent

a, FACS gating strategy (left) and DNA content histograms (right) of wild type (WT) and *TOP2B*$^{-/-}$ human RPE-1 cells under asynchronous (10% FCS) and serum-deprived (0% FCS) conditions, as measured by FACS following propidium iodide (PI) staining. G1, S and G2 populations are clearly present under asynchronous growing conditions. A strong G1 arrest is observed in serum deprived conditions. Percentages of cells in each of the indicated regions (red dotted brackets) are given.

b, Western blots demonstrating the absence of TOP2β protein in serum deprived and asynchronous (Asyn.) *TOP2B*$^{-/-}$ RPE-1 cells (left), and the absence of TOP2α in serum deprived wild type and *TOP2B*$^{-/-}$ RPE-1 cells (right). Ponceau S total protein loading is presented (Pon. S) for the left and right panels, and additionally a Ku80 loading control is included for the right panel.

c, Immunofluorescence experiment demonstrating induction of γ-H2AX foci (green) in asynchronous (Async.) and serum-deprived (Ser. Dep.) wild type (WT) and *TOP2B*$^{-/-}$ RPE-1 cells, all co-stained with DAPI (blue). Galleries of nine cells per condition were chosen randomly using Olympus ScanR Analysis software.

d, Quantification of (**c**). Numbers of γ-H2AX foci per cell were counted automatically using Olympus ScanR Analysis software. The mean ±SEM is reported for the indicated number of biological replicate experiments. Dots show the mean foci number per cell for individual replicate experiments.

e, Broad-scale *H. sapiens* TOP2 CC-seq maps in asynchronous and serum-deprived wild type (WT) and *TOP2B*$^{-/-}$ RPE-1 cells -VP16 (orange) and +VP16 (green). Raw hits on Watson and Crick strands were summed and smoothed according to local signal density. Source data are provided as a Source Data file.

Supplementary Figure 8

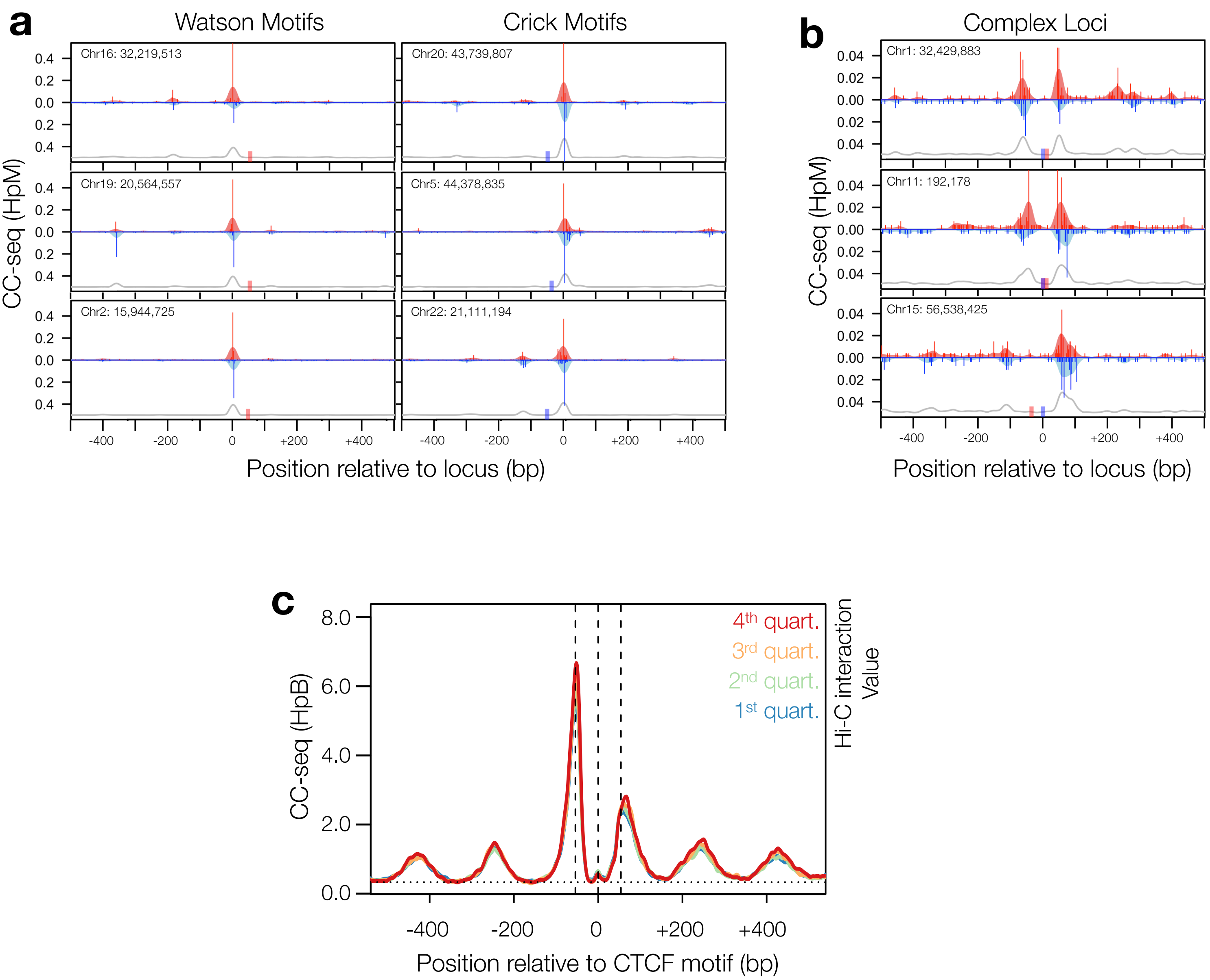

Supplementary Figure 8. TOP2 CC-seq signal enrichment around CTCF sites

a, Fine-scale TOP2 CC-seq maps of CTCF-proximal loci in human RPE-1 cells +VP16. Red and blue traces indicate TOP2-linked 5' DNA termini on the Watson and Crick strands, respectively. Pale shaded areas are the same data smoothed with a sliding 11 bp Hanning window. Red and Blue rectangles indicate the positions of CTCF motifs on the Watson and Crick strands respectively. The grey line indicates Hanning-smoothed sum of Watson and Crick TOP2 CC-seq signal.

b, Fine-scale mapping of TOP2 CCs surrounding three complex CTCF loci, processed as in (**a**).

c, Aggregation of TOP2 CCs in a 1 Kbp window centred on the subset of orientated CTCF motifs that can be assigned to a chromatin loop anchor in human RPE-1 cells[47]. Motifs are stratified into four quartiles of loop anchor interaction strength, and the average TOP2 CC distribution in each quartile plotted.

Supplementary Figure 9

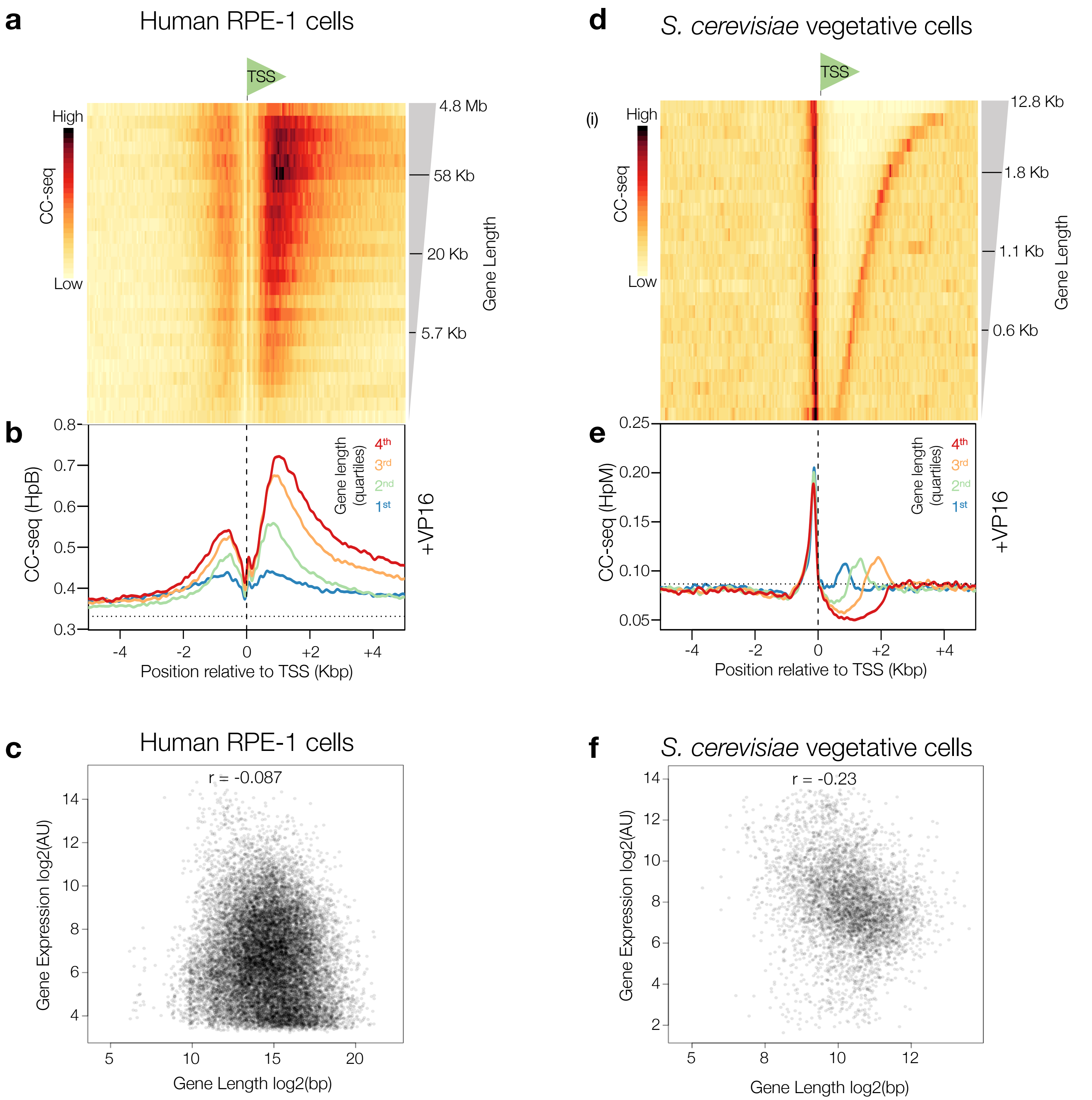

Supplementary Figure 9. TSS-proximal TOP2-linked DNA breaks in human cells, but not in *S. cerevisiae*, are correlated with gene length

a-b, Aggregation of TOP2 CCs in a 10 Kbp window centred on orientated TSSs in human RPE-1 cells. **a**, A heatmap of all TSSs in the human genome, with 25 aggregated rows stratified by gene length. The colour scale indicates average TOP2 CC density in 50 bp bins. **b,** Motifs are also stratified into quartiles of gene length, and the average TOP2 CC distribution in each quartile plotted.

c, Scatter plot of gene length versus gene expression, indicating no correlation (Pearson, r), suggesting that the observed relationship between CC-seq and gene length is not due to a relationship between gene length and gene expression.

d-f, As for (**a-c**), but *pdr1*Δ *S. cerevisiae* vegetative Top2 CC-seq samples and vegetative gene expression data[40].

Supplementary Figure 10

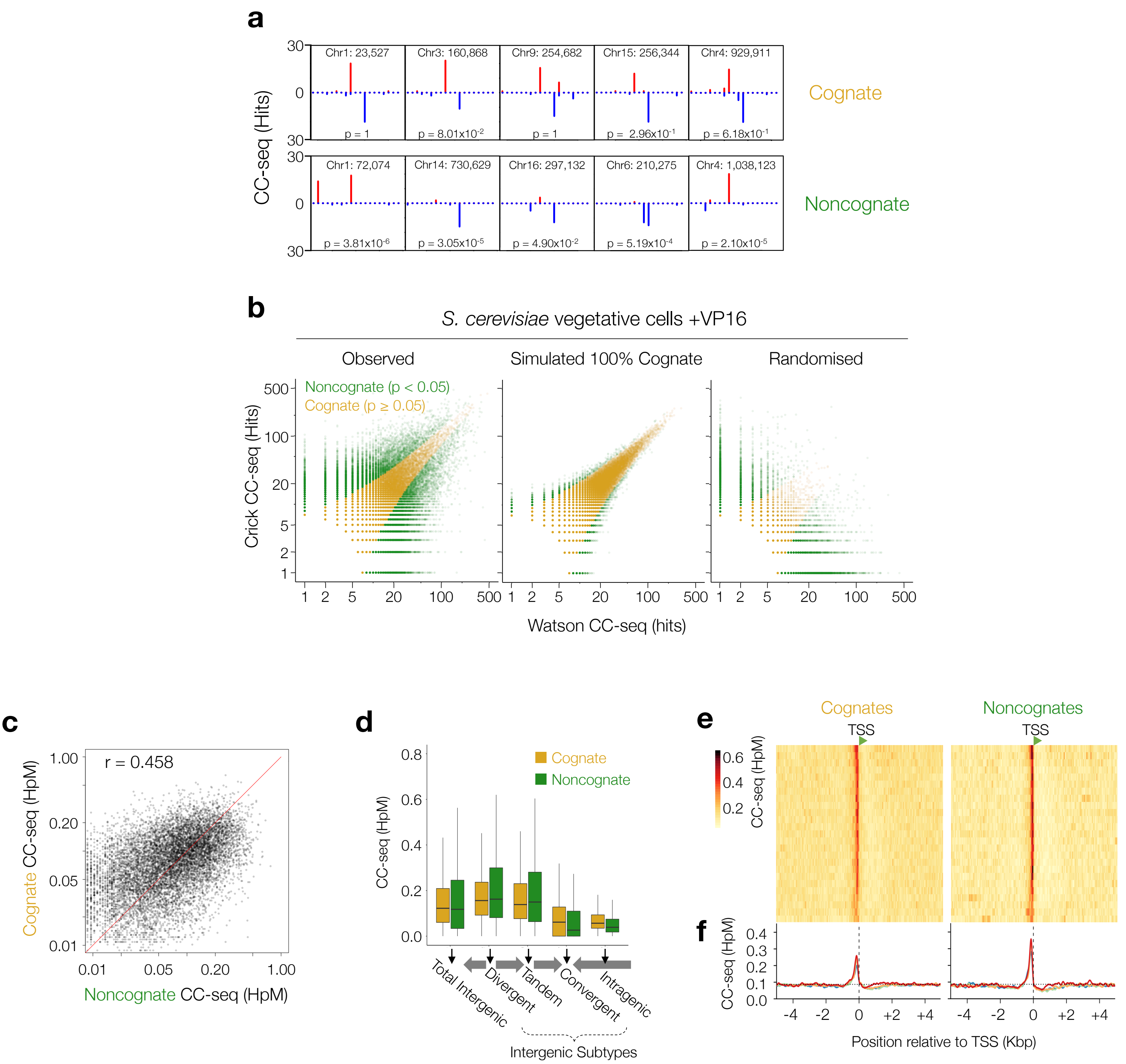

Supplementary Figure 10. Cognate and noncognate CC-seq signal is well-correlated at broad-scale in *S. cerevisiae.*

a, Examples of cognate and noncognate sites (with locations) in VP16-treated *S. cerevisiae pdr1Δ mre11Δ* cells. The p-value of the two-sample, two-tailed Poisson test between hits on Watson and Crick is given.

b, Scatterplots of Top2 CC-seq hits on the Watson strand vs the Crick strand at each 3 bp offset site, in the observed *S. cerevisiae pdr1Δ mre11Δ* +VP16 dataset (left); a dataset sampled from a simulated dataset where all 3 bp offset sites had identical Watson and Crick values; (centre); a randomised dataset where all observed Crick hits were shuffled (right). Noncognates (green) and cognates (gold) were defined as those 3 bp offset sites with significantly different, or not significantly different Watson and Crick hits, respectively (two-sample, two-tailed Poisson test; $p \leq 0.05$). Sites with fewer than 8 hits in total (Watson plus Crick) were excluded, due to insufficient statistical power.

c, Correlation (and Pearson r value) of 1 Kbp binned noncognate versus cognate Top2 CC-seq signal from VP16-treated *pdr1Δ mre11Δ S. cerevisiae*.

d, Quantification of cognate (gold) and noncognate (green) Top2 CC-seq signal in VP16-treated *pdr1Δ mre11Δ S. cerevisiae*, stratified by genomic region as in **Fig. 2d**.

e-f, Aggregation of cognate (left) or noncognate (right) Top2 CC-seq signal in a 10 Kbp window centred on orientated transcription start sites (TSS), in VP16-treated *pdr1Δ mre11Δ S. cerevisiae*. Heatmap of all TSSs in the *S. cerevisiae* genome, with 25 aggregated rows stratified by gene expression level[47] in vegetative growth. Colour scale indicates average Top2 CC-seq signal density in each 50 bp bin (**e**). Motifs are also stratified into quartiles of gene expression, and the average distribution of Top2 CC-seq signal in each quartile plotted (**f**).

HpM = Hits per million mapped reads per base pair.

Supplementary Figure 11

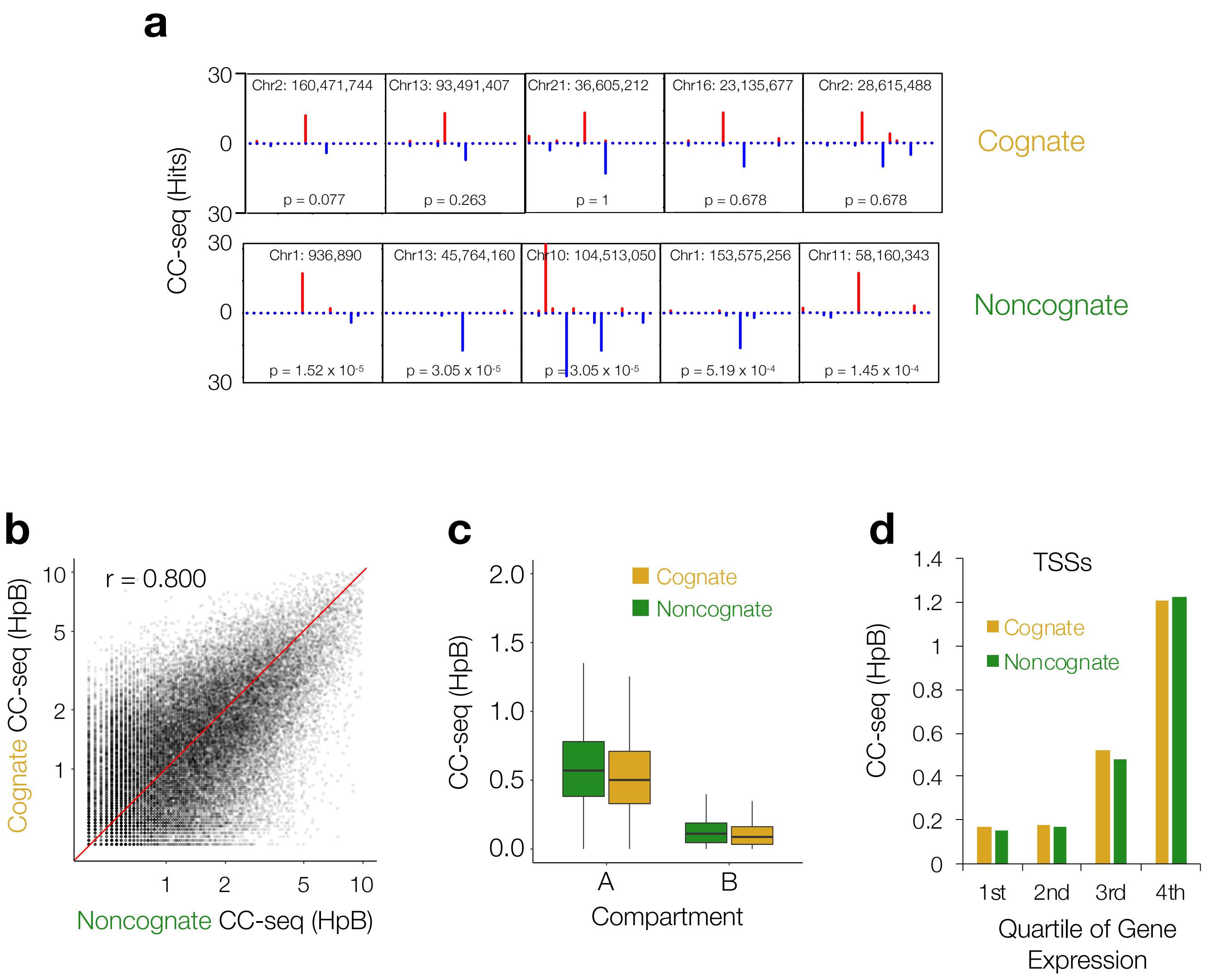

Supplementary Figure 11. Cognate and noncognate CC-seq signal is well-correlated at broad-scale in human cells.

a, Examples of cognate and noncognate sites (with locations) in VP16-treated human RPE-1 cells. The p-value of the two-sample, two-tailed Poisson test between hits on Watson and Crick is given.

b, Correlation scatterplot (and Pearson r value) of 10 Kbp binned noncognate and cognate TOP2 CC-seq signal from VP16-treated human RPE-1 cells.

c, Quantification of CC-seq cognate (gold) and noncognate (green) TOP2 CCs in chromatin compartments A and B. Data are expressed as box-and-whisker plots of density as for **Fig. 2d**

d, Mean cognate (gold) and noncognate (green) TOP2 CC-seq signal in a 10 Kbp window centred on TSSs stratified by gene expression level. Data were corrected using a background density equal to unfractionated CC-seq signal found in the genomic region not occupied by TSSs nor CTCF-bound CTCF motifs.

HpB = Hits per billion mapped reads per base pair.

Supplementary Figure 12

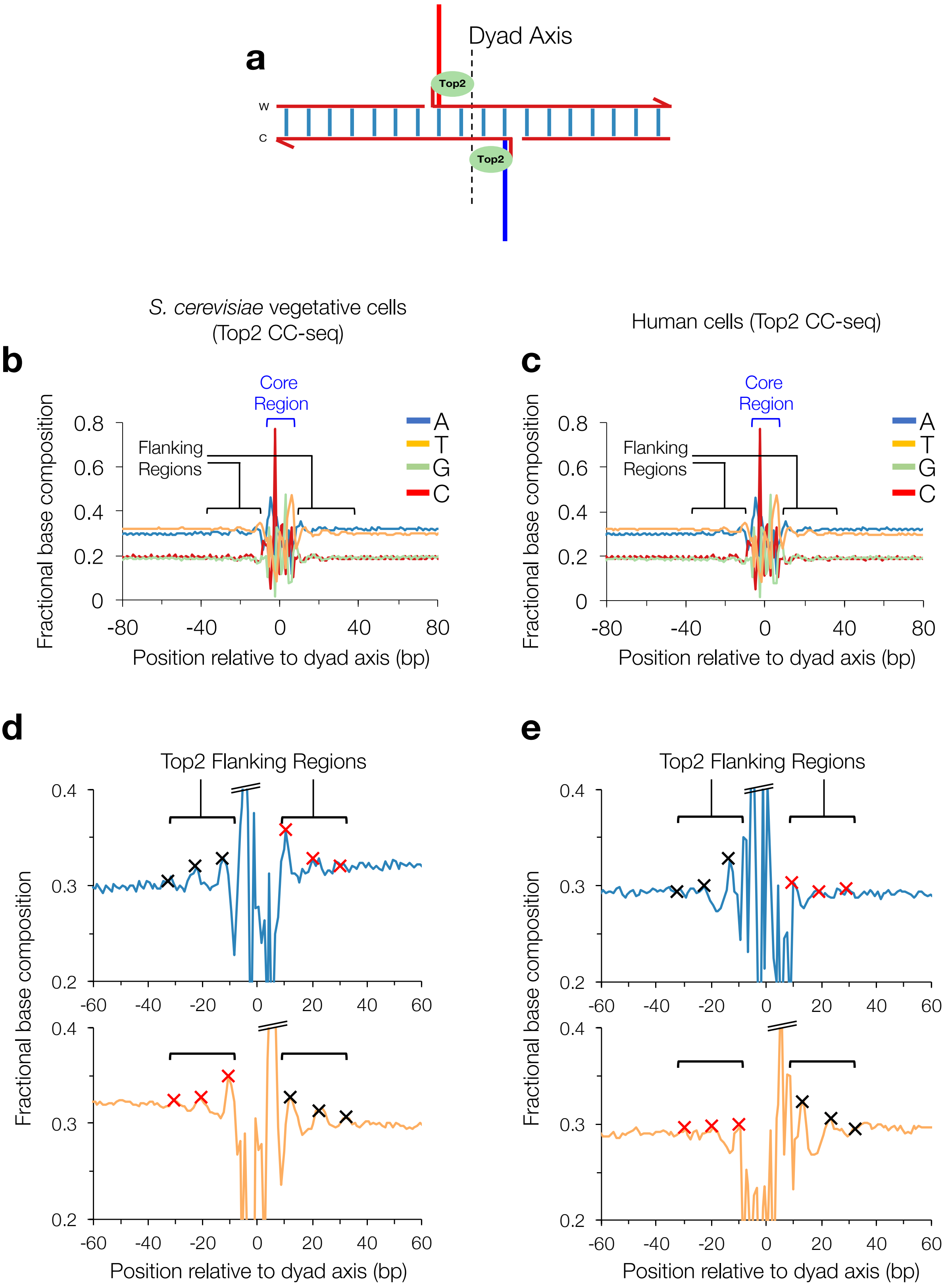

Supplementary Figure 12. Top2-linked DNA breaks in *S. cerevisiae* and human cells have nucleotide skews in core binding region, and in flanking regions indicative of DNA distortion.

a, Cartoon depicting the inferred position of the DSB dyad axis, relative to positions of Watson (red) and Crick (blue) hits.

b, Average nucleotide composition over a 160 bp window centred on the dyad axis in VP16-treated *pdr1Δ S. cerevisiae*. The position of the core region of bias (blue) and flanking regions of weaker bias (black) are indicated.

c, As in (**b**), but for the VP16-treated human RPE-1 dataset.

d-e, As in (**b-c**), but focussed in a 120 bp region centred on the dyad axis with A and T based separated for clarity. Top2 flanking regions displaying the weak ~10.5 bp periodic biases in nucleotide composition are highlighted with black and red crosses to emphasise the rotational symmetry of the skews.

Supplementary Figure 13

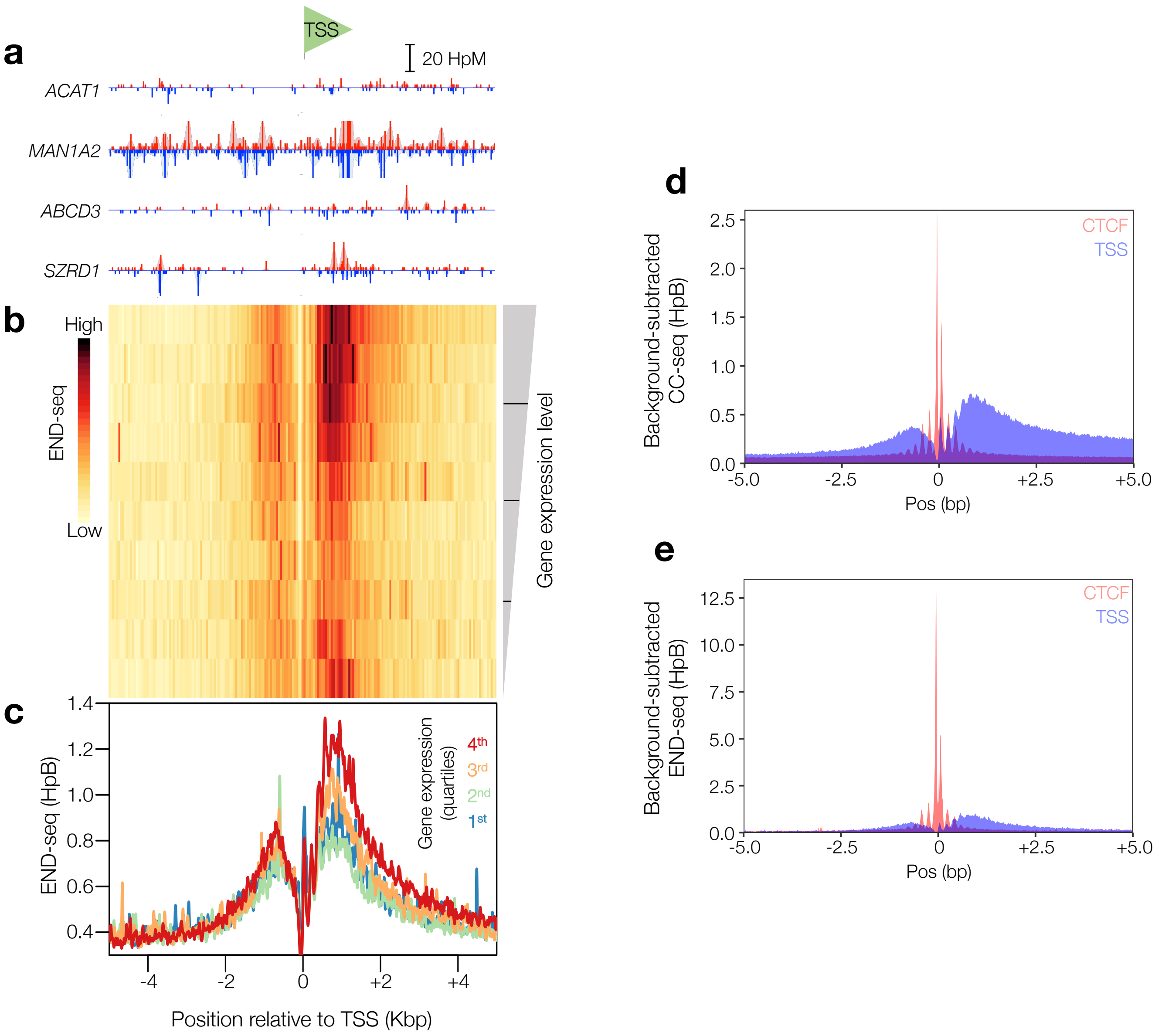

Supplementary Figure 13. TOP2 CC-seq signal enrichment around CTCF and TSS sites, compared with END-seq DSB signal
a-c, Aggregation of END-Seq DSBs in a 10 Kbp window centred on orientated TSSs in human MCF7 cells. Four example TSSs are shown orientated in the 5'-3' direction. Red and blue traces indicate DSB 5' DNA termini on the Watson and Crick strands, respectively. Pale shaded areas are the same data smoothed with a sliding 11 bp Hanning window as in **Fig. 5a**. (**a**). A heatmap of all TSSs in the human genome, with 25 aggregated rows stratified by MCF7 gene expression level[78]. The colour scale indicates average END-seq DSB density in each 50 bp bin (**b**). Motifs are also stratified into quartiles of gene expression, and the average END-seq DSB distribution in each quartile plotted. Horizontal line is the genome mean. Vertical line is the TSS (**c**).
d-e, Comparison of CTCF-proximal (pink) and TSS-proximal (blue) TOP2 CC-seq distributions (d) and END-seq DSB distributions (e) in the top quartile of CTCF-binding and gene-expression, respectively. Data were background-corrected by subtraction of average signal found in the genomic region containing neither TSSs, nor CTCF-bound CTCF motifs. The overlay provides a comparison of the relative strength and distribution of CTCF-proximal and TSS-proximal signals identified by the two techniques, and it does not indicate that CTCF sites and TSS sites are genomically colocalised.
HpB = Hits per billion mapped reads per base pair.

Supplementary Tables

Supplementary Table 1. DNA libraries used for mapping Spo11 activity. The number of reads remaining after each stage of the data pipeline are indicated.

Species	Strain/ Cell line	Condition	NGS Platform	Library code	Total Read Pairs	Mapped reads	Blacklist-filtered reads	% of Total
S. cerevisiae	*sae2*Δ	Meiosis	MiSeq	1	3512089	3376630	3177210	90.5
				2A	10101423	8796998	8564017	84.8
				3	4957477	4745192	4502308	90.8
				5	4012972	3705100	3559541	88.7
				7BC	4065600	3779390	3569290	87.8

Supplementary Table 2. DNA libraries used for mapping Top2 activity. The number of reads remaining after each stage of the data pipeline are indicated.

Species	Strain/ Cell line	Condition	NGS Platform	Library code	Total Read Pairs	Mapped reads	De-duplicated reads	Blacklist-filtered reads	% of Total	Pooled Reads (Millions)
S. cerevisiae	*pdr1Δ*	-VP16	NextSeq 500	RA1	3741251	3542955	3179049	2746294	73.4	3.50
			MiSeq	RA8	1118483	879107	842290	751850	67.2	
S. cerevisiae	*pdr1Δ*	+VP16	NextSeq 500	RA2	3096257	3053356	2679303	2234941	72.2	3.93
			MiSeq	RA9	2262958	2071231	1958341	1690075	74.7	
S. cerevisiae	*pdr1Δ sae2Δ*	-VP16	NextSeq 500	RA4	3634284	3552818	3215523	2745668	75.5	3.79
			MiSeq	RA10	1420662	1214067	1181399	1046827	73.7	
S. cerevisiae	*pdr1Δ sae2Δ*	+VP16	NextSeq 500	RA5	2529368	2476714	1386551	1097282	43.4	3.54
			MiSeq	RA11	3226626	3063388	2824882	2444486	75.8	
S. cerevisiae	*pdr1Δ mre11Δ*	-VP16	NextSeq 500	RA6	5345587	5205411	4288662	3646842	68.2	4.76
			MiSeq	RA12	1519020	1326435	1280137	1109310	73.0	
S. cerevisiae	*pdr1Δ mre11Δ*	+VP16	NextSeq 500	RA7	3382546	3357168	2921333	2526342	74.7	5.54
			MiSeq	RA13	4133347	4032996	3540366	3010542	72.8	
Human	RPE-1 WT	-VP16	NextSeq 500	WG11	125203096	109503841	68845619	68639594	54.8	185.36
				WG15	125328792	110432447	46588296	46449378	37.1	
				WG23	89154181	77728045	70484665	70269334	78.8	
Human	RPE-1 WT	+VP16	NextSeq 500	WG12	110910655	95546007	55848200	55681939	50.2	276.97
				WG16	102804692	91872706	54312084	54164650	52.7	
				WG24	130816235	114086421	101162102	100857557	77.1	
				WG34	91829516	79751337	66458538	66270349	72.2	
Human	RPE-1 WT	-VP16 G1	MiSeq	WG13	2601642	2292863	2007177	1991088	76.5	4.27
				WG21	2610523	2298289	2295625	2277124	87.2	
Human	RPE-1 WT	+VP16 G1	MiSeq	WG14	3075620	2771819	2491674	2475350	80.5	5.23
				WG22	3139164	2779809	2772349	2751281	87.6	
Human	RPE-1 WT	-VP16 Async	MiSeq	WG15b*	3207507	2866283	2782876	2760652	86.1	5.35
				WG23	2959289	2616954	2608670	2586523	87.4	
Human	RPE-1 WT	+VP16 Async	MiSeq	WG16b*	3018163	2721770	2673273	2656580	88.0	6.30
				WG24	4165048	3682708	3668602	3639324	87.4	
Human	RPE-1 TOP2B$^{-/-}$	-VP16 G1	MiSeq	WG17	2978044	2624262	2558332	2536752	85.2	4.69
				WG25	2460949	2181002	2173777	2155101	87.6	
Human	RPE-1 TOP2B$^{-/-}$	+VP16 G1	MiSeq	WG18	2927745	2588870	2557532	2537085	86.7	5.10
				WG26	2922624	2596547	2587152	2565454	87.8	
Human	RPE-1 TOP2B$^{-/-}$	-VP16 Async	MiSeq	WG19	3713068	3278164	3188049	3162895	85.2	7.04
				WG27	4413242	3943962	3910035	3877611	87.9	
Human	RPE-1 TOP2B$^{-/-}$	+VP16 Async	MiSeq	WG20	3426100	3038601	2978047	2956076	86.3	8.13
				WG28	5878342	5251089	5211027	5169010	87.9	

Supplementary Table 3. *S. cerevisiae* strains used in this study. Strains MJ315 and M319 (both diploid SK1 background) were used for meiotic (Spo11) CC-seq mapping; strains MJ429, MJ475 and MJ551 (BY4741 haploid background) were used for vegetative (Top2) CC-seq mapping.

Strain	Number	Genotype
sae2Δ	MJ315	SK1: *ho::LYS2/', lys2/', ura3/', arg4-nsp/', leu2::hisG/', his4X::LEU2/', nuc1::LEU2/', sae2Δ::KanMX6/'*
sae2Δ spo11-Y135F	MJ319	SK1: *ho::LYS2/', lys2/', ura3/', arg4-nsp/', leu2::hisG/', his4X::LEU2/', nuc1::LEU2/', spo11(Y135F)-HA3His6::KanMX4/', sae2Δ::KanMX6/'*
pdr1Δ	MJ429	BY4741: *ura3Δ0, leu2Δ0, his3Δ1, met15Δ0, pdr1Δ::PDR1-DBD-CYC8::LEU2*
pdr1Δ sae2Δ	MJ475	BY4741: *ura3Δ0, leu2Δ0, his3Δ1, met15Δ0, pdr1Δ::PDR1-DBD-CYC8::LEU2, sae2Δ::KanMX6*
pdr1Δ mre11Δ	MJ551	BY4741: *ura3Δ0, leu2Δ0, his3Δ1, met15Δ0, pdr1Δ::PDR1-DBD-CYC8::LEU2, mre11Δ::KanMX4*

Supplementary Table 4. Human cell lines used in this study.

Parent cell line	Clone number	Genotype
hTERT RPE-1	WT	WT
hTERT RPE-1	T2B/1	$TOP2B^{-/-}$

Supplementary Table 5. The annealed structures of the custom P5 and P7 adapters. "T_i" indicates an inverted dT, linked via a 3'-3' phosphodiester. This inhibits unwanted ligation at this end of the adapter. Also note that the 5'-terminal moieties of each adapter (A for P5, G for P7) are nucleosides (3'-OH), which further inhibits ligation at this end

Adapter	Annealed Structure
P5	blocked end ACACTCTTTCCCTACACGACGCTCTTCCGATCT / T_iTGTGAGAAAGGGATGTGCTGCGAGAAGGCTAGp ligated end
P7	ligated end pGATCGGAAGAGCACACGTCTGAACTCCAGTCACT$_i$ / TCTAGCCTTCTCGTGTGCAGACTTGAGGTCAGTG blocked end

Supplementary Table 6. Publicly available datasets used in this study.

Species/ Cell line	Dataset Type	Accession(s)	Reference
S. cerevisiae / SK1 (meiotic)	Gene expression microarray	GSM907178, GSM90719, GSM907180	40
S. cerevisiae / SK1 (vegetative)	Gene expression microarray	GSM907176, GSM907177	40
S. cerevisiae / SK1 (vegetative)	oligo-seq	GSE26449	28
S. cerevisiae SK1 (vegetative)	MNase-seq	GSM1849301, GSM1849302	37
S. cerevisiae SK1 (meiotic)	MNase-seq	GSM1424408	37
S. cerevisiae SK1 (meiotic)	CAGE	http://www.yeastss.org/download/	39
H. sapiens / RPE-1	Gene expression microarray	GSM1395252, GSM1395253, GSM1395254	48
H. Sapiens / MCF7	Gene expression microarray	GSM1141244	78
H. sapiens / RPE-1	Hi-C	GSE71831	47
H. sapiens / RPE-1	bTMP-seq	GSM1062645, GSM1062646	44
H. sapiens / MCF7	END-seq	GSM2635568	18
H. sapiens / RPE-1	CTCF ChIP-seq	GSM749673, GSM1022665	71
H. sapiens / RPE-1	H3K4Me3 ChIP-seq	GSM945271, GSM945271	71
H. sapiens / RPE-1	H3K27Ac ChIP-seq	GSM733771, GSM733718, GSM733755, GSM733691, GSM733656, GSM733674, GSM733646	71
H. sapiens / RPE-1	Methyl-RRBS	GSM683773, GSM683905	71
H. sapiens / K562	MNase-seq	GSM920557	71
H. sapiens / ARPE	GRO-seq	GSM2428724	50
H. sapiens / RPE	CAGE	DRA000991	49
