# Supplementary material for: A nucleotide resolution map of Top2-linked DNA breaks in the yeast and human genome
Source: Nat Commun. 2019 Oct 24;10:4846. doi: 10.1038/s41467-019-12802-5 (PMC6813358; doi:10.1038/s41467-019-12802-5)
Supplement: Supplementary file 3 — Reporting Summary [file 41467_2019_12802_MOESM3_ESM.pdf]

nature research

Corresponding author(s): Matthew J Neale; William H Gittens

Last updated by author(s): Jul 24, 2019

Reporting Summary

Nature Research wishes to improve the reproducibility of the work that we publish. This form provides structure for consistency and transparency in reporting. For further information on Nature Research policies, see Authors & Referees and the Editorial Policy Checklist.

Statistics

For all statistical analyses, confirm that the following items are present in the figure legend, table legend, main text, or Methods section.

n/a	Confirmed	
☐	☒	The exact sample size (*n*) for each experimental group/condition, given as a discrete number and unit of measurement
☐	☒	A statement on whether measurements were taken from distinct samples or whether the same sample was measured repeatedly
☐	☒	The statistical test(s) used AND whether they are one- or two-sided<br>*Only common tests should be described solely by name; describe more complex techniques in the Methods section.*
☒	☐	A description of all covariates tested
☐	☒	A description of any assumptions or corrections, such as tests of normality and adjustment for multiple comparisons
☐	☒	A full description of the statistical parameters including central tendency (e.g. means) or other basic estimates (e.g. regression coefficient) AND variation (e.g. standard deviation) or associated estimates of uncertainty (e.g. confidence intervals)
☐	☒	For null hypothesis testing, the test statistic (e.g. *F*, *t*, *r*) with confidence intervals, effect sizes, degrees of freedom and *P* value noted<br>*Give P values as exact values whenever suitable.*
☒	☐	For Bayesian analysis, information on the choice of priors and Markov chain Monte Carlo settings
☒	☐	For hierarchical and complex designs, identification of the appropriate level for tests and full reporting of outcomes
☐	☒	Estimates of effect sizes (e.g. Cohen's *d*, Pearson's *r*), indicating how they were calculated

Our web collection on statistics for biologists contains articles on many of the points above.

Software and code

Policy information about availability of computer code

Data collection	N/A
Data analysis	Open source software available here: https://github.com/Neale-Lab<br>Other data analysis performed using R (v3.5)

For manuscripts utilizing custom algorithms or software that are central to the research but not yet described in published literature, software must be made available to editors/reviewers. We strongly encourage code deposition in a community repository (e.g. GitHub). See the Nature Research guidelines for submitting code & software for further information.

Data

Policy information about availability of data

All manuscripts must include a data availability statement. This statement should provide the following information, where applicable:

- Accession codes, unique identifiers, or web links for publicly available datasets
- A list of figures that have associated raw data
- A description of any restrictions on data availability

The source data underlying Figs 1b, 2a, 3a and Supplementary Figs S7a-d are provided as a Source Data file. CC-seq data used in Figs 1b-d, 2b-f, 3b-f, 4a-f, 5a-j, 6a-f and Supplementary Figs S2a-e, S3a-k, S4a-d, S5a-j, S6a-e, S7e, S8a-c, S9a-f, S10a-f, S11a-d, S12b-e, and S13d have been deposited in the NCBI GEO database under accession numbers (Pending). Raw unmapped FASTQ data underlying the same figures have been deposited in the NCBI SRA database under the accession numbers SRP186470 (S. cerevisiae Spo11), SRP186446 (S. cerevisiae Top2), SRP187576 (human Top2), All strains and cell lines listed in Table S3 and S4 are available from the corresponding author upon request.

Field-specific reporting

Please select the one below that is the best fit for your research. If you are not sure, read the appropriate sections before making your selection.

☒ Life sciences ☐ Behavioural & social sciences ☐ Ecological, evolutionary & environmental sciences

For a reference copy of the document with all sections, see nature.com/documents/nr-reporting-summary-flat.pdf

Life sciences study design

All studies must disclose on these points even when the disclosure is negative.

Sample size	All CC-seq libraries were prepared from unique biological replicates. Every condition was assayed at least in duplicate. High reproducibility was observed (see below).
Data exclusions	Human data sets were filtered to remove ultra-high signal regions{Hoffman et al., 2013; ENCODE, 2012} and repeat regions{ENCODE, 2012}, which are blacklisted. Yeast data sets were filtered to exclude highly repetitive regions: long terminal repeats, retrotransposons, telomeres, and the rDNA.
Replication	High reproducibility was observed between replicates of each condition (see Figs S2b, S3a-d, and S6a-b).
Randomization	Not relevant to this study.
Blinding	Investigators were not blinded because none of the assays that we employed required human selection/scoring (e.g manual IF foci scoring).

Reporting for specific materials, systems and methods

We require information from authors about some types of materials, experimental systems and methods used in many studies. Here, indicate whether each material, system or method listed is relevant to your study. If you are not sure if a list item applies to your research, read the appropriate section before selecting a response.

Materials & experimental systems

n/a	Involved in the study	
☐	☒	Antibodies
☐	☒	Eukaryotic cell lines
☒	☐	Palaeontology
☒	☐	Animals and other organisms
☒	☐	Human research participants
☒	☐	Clinical data

Methods

n/a	Involved in the study	
☒	☐	ChIP-seq
☐	☒	Flow cytometry
☒	☐	MRI-based neuroimaging

Antibodies

Antibodies used	TOP2β (Clone 40, BD Biosciences), TOP2α (ab52934, Abcam), or Ku80 (ab80592, Abcam), HRP-conjugated Rabbit anti-Mouse IgG (ThermoFisher), Alexa 488-conjugated Goat anti-mouse IgG (Fisher), and Alexa 647-conjugated Goat anti-Rabbit (Fisher)
Validation	TOP2β (Clone 40, BD Biosciences): "This antibody is routinely tested by Western blot analysis and immunofluorescent imaging. Other applications were tested at BD Biosciences Pharmingen during antibody development only. Human (QC Testing)."<br><br>TOP2α (ab52934, Abcam): "Suitable applications: WB, IP, IH-C in Mouse, Rat and Human"<br><br>Ku80 (ab80592, Abcam): "Suitable applications: WB, IP, IHC-P, ICC/IFF, Flow Cyt in Human"<br><br>HRP-conjugated Rabbit anti-Mouse IgG (ThermoFisher): "Application(s): IHC, indirect ELISA, WB"<br><br>Alexa 488-conjugated Goat anti-mouse IgG (Fisher): "Tested Applications: Flow Cy, ICC, IF"<br><br>Alexa 647-conjugated Goat anti-Rabbit (Fisher): "Application: ELISA, Flow Cyt, ICC/IF, IHC-Fr, IHC-P"

Eukaryotic cell lines

Policy information about cell lines

Cell line source(s)	RPE-1: ATCC
Authentication	ATCC Authentication
Mycoplasma contamination	All cell lines tested negative for mycoplasma (in-house testing).
Commonly misidentified lines (See ICLAC register)	None

Flow Cytometry

Plots

Confirm that:

☒ The axis labels state the marker and fluorochrome used (e.g. CD4-FITC).

☒ The axis scales are clearly visible. Include numbers along axes only for bottom left plot of group (a 'group' is an analysis of identical markers).

☒ All plots are contour plots with outliers or pseudocolor plots.

☒ A numerical value for number of cells or percentage (with statistics) is provided.

Methodology

Sample preparation	Approximately 10 million RPE-1 cells were trypsinised, washed once in PBS and resuspended in 1.5 ml PBS. 3.5 ml ethanol was added dropwise to pellet, with vortexing. Cells were fixed for 1 hr at 4 ˚C, prior to centrifugation and aspiration of the supernatant. Cells were washed twice with PBS, prior to resuspension in 0.5 ml 0.25% Triton-X100-PBS for 15 min on ice. Cells were pelleted by centrifugation, supernatant was aspirated, and the pellet was resuspended in 0.5 ml TBS containing 10 ug/ml RNase A (Sigma) and 167 nM Sytox Green (ThermoFisher). After 30 min incubation in the dark at RT, the suspension was filtered through fine mesh into test tubes. DNA content in 50,000 cells was analysed using the Accuri C6 (BD Biosciences), with gating to exclude doublets and cell debris.
Instrument	Accuri C6 (BD Biosciences)
Software	BD CSampler
Cell population abundance	RPE-1 WT Asynchronous: 78,264<br>RPE-1 WT G1 : 79,545<br>RPE-1 TOP2B-/- Asynchronous: 74,287<br>RPE-1 TOP2B-/- G1: 71,588
Gating strategy	Gating to remove doublets and cell debris based on SSC-A vs SSC-H

☒ Tick this box to confirm that a figure exemplifying the gating strategy is provided in the Supplementary Information.
